# Increased oxidative stress contributes to enhance brain amyloidogenesis and blunts energy metabolism in sucrose-fed rat: effect of AMPK activation

**DOI:** 10.1038/s41598-021-98983-w

**Published:** 2021-10-01

**Authors:** Luz Camacho-Castillo, Bryan V. Phillips-Farfán, Gabriela Rosas-Mendoza, Aidee Baires-López, Danira Toral-Ríos, Victoria Campos-Peña, Karla Carvajal

**Affiliations:** 1 Laboratorio de Nutrición Experimental, Instituto Nacional de Pediatría, Insurgentes Sur 3700 C, Col. Insurgentes Cuicuilco, Del. Coyoacán, 04530 CD Mexico, Mexico; 2 Laboratorio Experimental de Enfermedades Neurodegenerativas, Instituto Nacional de Neurología y Neurocirugía “Manuel Velasco”, CD México, México

**Keywords:** Neuroscience, Metabolism, Biochemistry, Kinases, Experimental models of disease

## Abstract

Metabolic disturbances are linked to neurodegenerative diseases such as Alzheimer disease (AD). However, the cellular mechanisms underlying this connection are unclear. We evaluated the role of oxidative stress (OS), during early metabolic syndrome (MetS), on amyloidogenic processes in a MetS rat model induced by sucrose. MetS caused OS damage as indicated by serum and hypothalamus lipid peroxidation and elevated serum catalase activity. Tissue catalase and superoxide dismutase activity were unchanged by MetS, but gene expression of nuclear factor erythroid-derived 2-like 2 (NFE2L2), which up-regulates expression of antioxidant enzymes, was higher. Expression of amyloid-β cleaving enzyme 1 (BACE-1) and amyloid precursor protein (APP), key proteins in the amyloidogenesis pathway, were slightly increased by sucrose-intake in the hippocampus and hypothalamus. Activation and expression of protein kinase B (PKB) and AMP-dependent protein kinase (AMPK), pivotal proteins in metabolism and energy signaling, were similarly affected in the hippocampus and hypothalamus of MetS rats. Brain creatine kinase activity decreased in brain tissues from rats with MetS, mainly due to irreversible oxidation. Chronic metformin administration partially reversed oxidative damage in sucrose-fed animals, together with increased AMPK activation; probably by modulating BACE-1 and NFE2L2. AMPK activation may be considered as a preventive therapy for early MetS and associated neurodegenerative diseases.

## Introduction

Metabolic syndrome (MetS) is defined as a cluster of biometric and biochemical factors -such as glucose intolerance, dyslipidemias, hypertension and obesity- that cause type II diabetes (T2D) and cardiovascular diseases^1^. Its underlying mechanisms likely include insulin resistance (IR, an inability to respond to the hormone stimulus) and cellular energy disturbances leading to an ATP deficit^2^. Together with insulin, the metabolic cascade orchestrated by adenosine monophosphate-dependent kinase (AMPK) plays an important role in controlling cellular energy balance, since AMPK is a metabolic sensor and key gauge of energy processes^3^. Disturbances in the insulin and AMPK pathways are tightly related to the development of MetS. AMPK is activated by the anti-diabetic drug metformin and may be responsible of its main therapeutic effects. Growing evidence strongly suggests a relationship between MetS and neurodegenerative diseases (ND). Accordingly, MetS can lead to development of severe illnesses such as Alzheimer´s disease (AD)^4,5^. IR may interfere with amyloid-β (Aβ) and tau metabolism causing neuritic plaque and neurofibrillary tangle formation, the major AD lesions^6^. In addition, epidemiological studies suggest that IR, T2D and obesity increase the risk of suffering ND^7^. Moreover, metformin also has been shown to reduce the risk of developing ND, senile dementia or AD^8,9^.

A derangement of energy homeostasis is associated to MetS and to increased production of reactive oxygen species (ROS)^10^, leading to sustained oxidative stress (OS). Many cellular and metabolic consequences of OS might induce the development of MetS and ND. In this line, genetically obese rats with IR bear marked cerebrovascular dysfunction associated with OS^11^. Moreover, it has been shown that OS affects the brain in AD; since increased nucleic acid oxidation, lipid peroxidation, protein oxidation, nytrosylation and carbonylation were observed. Indeed, this kind of damage may be caused by Aβ accumulation^12–14^.

To better understand how metabolic disturbances may contribute to ND related to AD, we investigated the participation of excessive ROS production and AMPK activation in an animal model of premature MetS. Wistar rats received 30% sucrose in their drinking water during 16 weeks. This widely validated paradigm^15,16^ induces metabolic disturbances including insulin resistance and hypertriglyceridemia, generally without hyperglycemia (fasting blood glucose) or weight gain^17–19^. We also studied the role of excessive ROS production and AMPK activation in modulating amyloid-β cleaving enzyme 1 activity (BACE-1) and amyloid precursor protein expression (APP), which are involved in the amyloidogenic pathway. We examined the insulin pathway and oxidative markers; such as lipid peroxidation, total antioxidant capacity, enzymatic antioxidant systems, the highly OS-sensitive brain creatine kinase (CK) and nuclear factor erythroid-derived 2-like 2. We evaluated whether activating AMPK had beneficial consequences on the deleterious metabolic effects of OS produced by MetS. These issues were studied in the hypothalamus and hippocampus given their important participation in MetS^20–22^ and AD^21,23,24^, respectively.

## Results

### Effect of MetS on biochemical and biometric values

**Figure 1:**
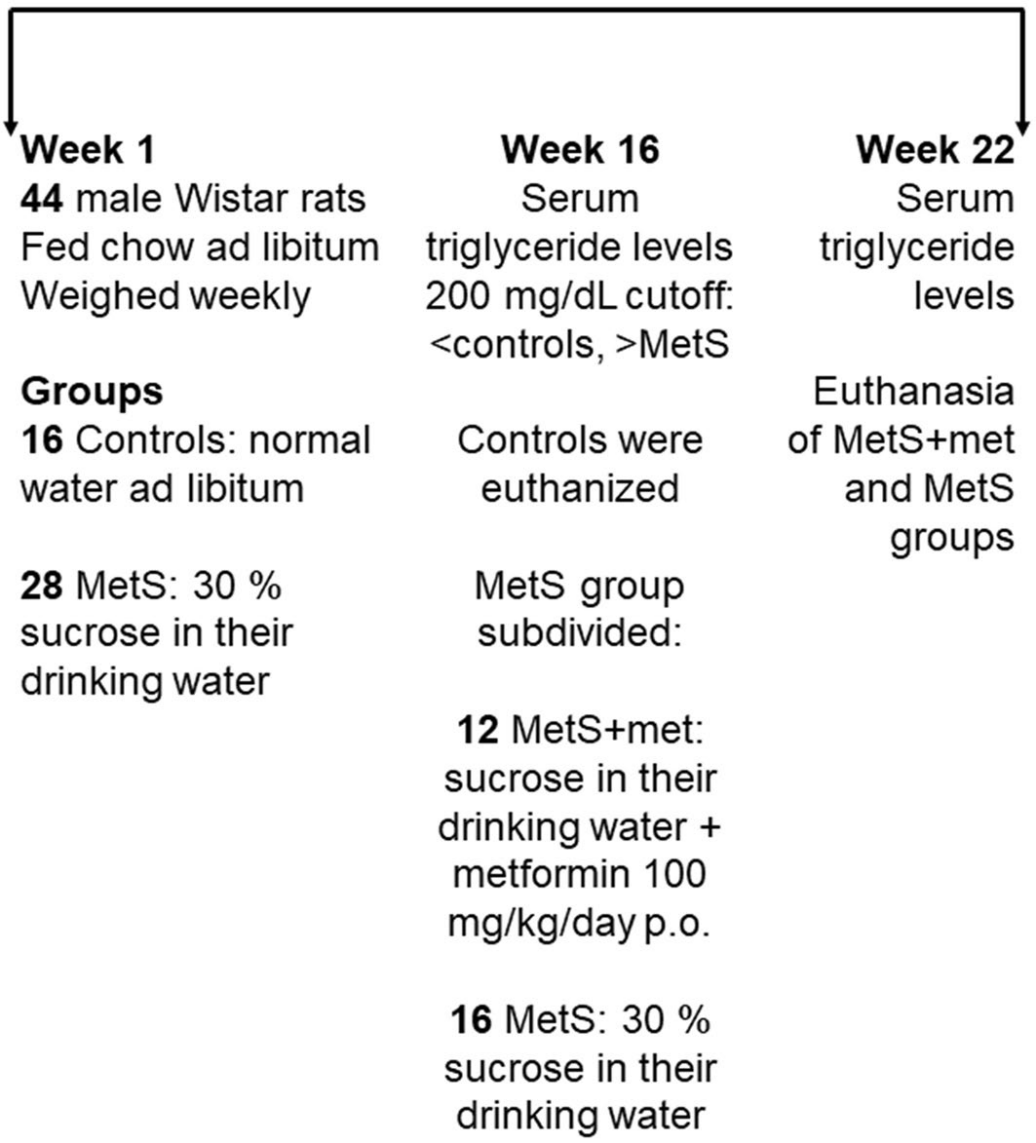
Timeline showing the experimental design with number of animals used (total and per group or subgroup) as well as the groups, treatments, measurement of serum triglyceride levels (which defined the inclusion into either the control or MetS groups) and animal euthanasia.

The sucrose diet induced changes on the biochemical and biometric variables measured (Table 1). It is important to note that control values were taken at week 16, before taking the values for other two groups at week 22 (the experimental design is shown in Fig. 1). As formerly reported, rats subjected to hyper-caloric sucrose regimens showed significantly augmented triglyceride and insulin levels^25,26^. The homeostatic modeling assessment (HOMA) index, used to assess IR, and their visceral fat were also notably elevated compared to controls. In fact, the fat/body mass ratio indicated severe obesity in these rats, although no significant changes in body weight were found. Metformin treatment during 6 weeks produced no effect on triglyceride levels, visceral fat accumulation or body weight of animals with MetS. On the contrary, metformin reduced glucose and insulin levels; meaningfully diminishing the HOMA index.

**Table 1:** Biochemical and biometric parameters.

Parameters	Control week 16, n = 16	MetS week 22, n = 16	MetS + metformin week 22, n = 8
Serum triglycerides (mg/dL)	167 ± 44	270 ± 92***	269 ± 67.9**
Body weight (g)	446 ± 60	466 ± 32	470 ± 45.3
Visceral fat (g)	10.6 ± 4.0	14.9 ± 6.0*	18.3 ± 5.1**
Visceral fat/body weight (%)	2.0 ± 1.6	3.3 ± 1.2**	3.9 ± 0.8^^
Insulin (ng/mL)	5.6 ± 1.6	9.2 ± 0.8***	6.2 ± 1.4^^^
Serum glucose (mg/dL)	132 ± 4.0	131 ± 8.0	116 ± 8.5***^^^
HOMA index	1.8 ± 0.4	2.8 ± 0.4***	1.9 ± 0.3^^^

The controls received standard chow and tap water for 16 weeks. Metabolic syndrome (MetS) was induced by 30% sucrose in the drinking water. The parameters were measured at the end of 16 weeks and after 6 more weeks with 30% sucrose (MetS) or 30% sucrose plus metformin (100 mg/kg/day p.o., MetS + metformin). The data represent the mean ± SD. *p ≤ 0.05 vs. control, **p ≤ 0.01 vs. control, ***p ≤ 0.001 vs. control; ^p ≤ 0.05 vs. MetS, ^^p ≤ 0.01 vs. MetS, ^^^p ≤ 0.001 vs. MetS.

### Effect of MetS and metformin administration on insulin and AMPK pathways

**Figure 2:**
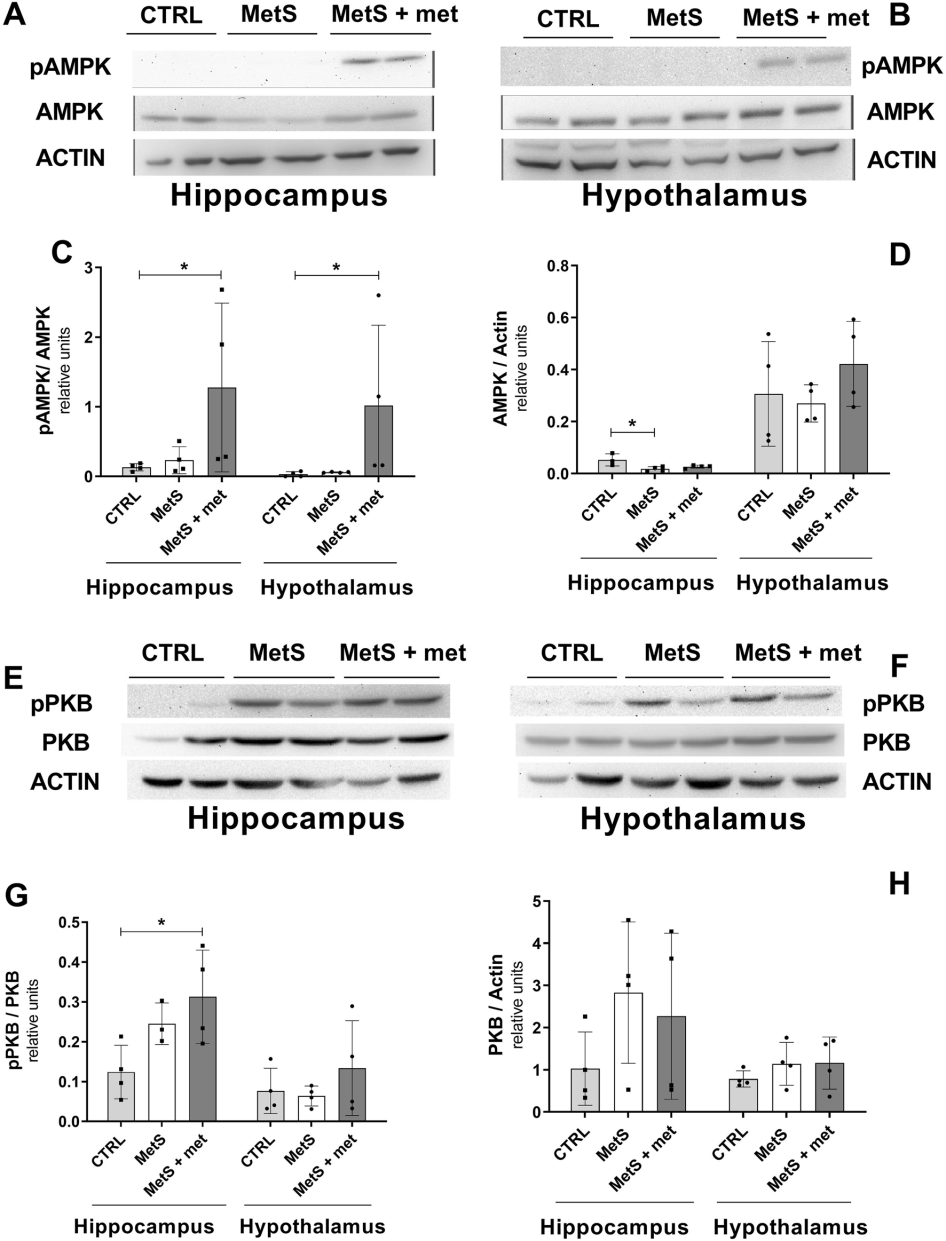
Effect of the metabolic syndrome (MetS) and metformin on AMP-dependent protein kinase (AMPK) and protein kinase B (PKB) phosphorylation and expression. (**A**, **B**) Representative blots showing phosphorylated and total AMPK. (**C**, **D**) Densitometric analysis of relative AMPK phosphorylation and total AMPK expression. (**E**, **F**) Representative blots showing phosphorylated and total PKB. (**G**, **H**) Densitometric analysis of relative PKB phosphorylation and total PKB expression. The data represent the mean ± SD. Sample size (N) CTRL = 4, MetS = 4, MetS + met = 4 for all except hippocampal AMPK expression (CTRL = 3) and PKB phosphorylation (MetS = 3). *p ≤ 0.05.

To explore the effect of MetS on brain energy metabolism, we evaluated the activation of two kinases controlling fuel balance: AMPK and protein kinase B (PKB/AKT; Fig. 2A,B,E,F). AMPK phosphorylation (Fig. 2C) and expression (Fig. 2D) were unchanged in the hypothalamus of rats with MetS, while there was a reduction of AMPK expression in the hippocampus. Metformin administration increased AMPK phosphorylation (Fig. 2C) in both brain areas relative to control rats, but did not change its expression (Fig. 2D) in the hippocampus or hypothalamus of MetS rats.

PKB/AKT phosphorylation (Fig. 2G) and expression (Fig. 2H) were unchanged in the hippocampus and hypothalamus of animals with MetS relative to controls. Metformin treatment increased PKB activation (Fig. 2G) in the hippocampus, but not in the hypothalamus of sucrose-fed animals. Metformin treatment did not alter total PKB expression (Fig. 2H) in either brain area.

### Effects of MetS and AMPK activation on neurodegenerative markers

We evaluated some neurodegenerative markers related to AD in the hippocampus and the hypothalamus of sucrose-fed rats to understand the contribution of MetS to neurodegenerative processes. Particularly, we evaluated Aβ cleaving enzyme-1 (BACE-1) and amyloid-β precursor protein (APP). Both proteins are highly involved in the formation of neuritic plaques^27^.

**Figure 3:**
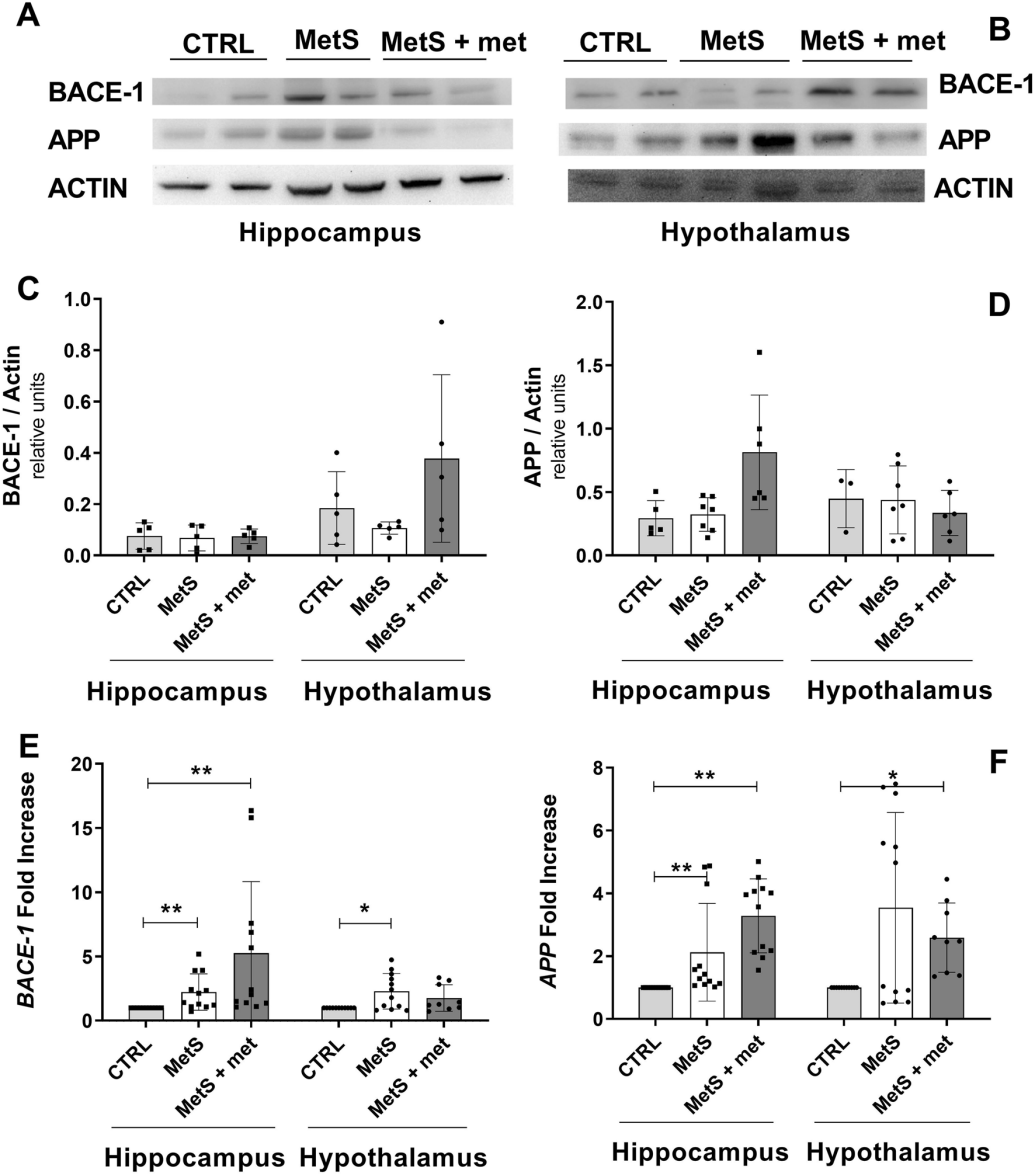
Effect of the metabolic syndrome (MetS) and metformin on total protein and gene expression of the amyloid-β cleaving enzyme (BACE)-1 and amyloid-β protein precursor (APP). (**A**, **B**) Representative blots showing expression of BACE-1 and APP. (**C**, **D**) Densitometric analysis of BACE-1 protein and APP protein expression. (**E**, **F**) *BACE-1* and *APP* gene expression. The data represent the mean ± SD. Sample size (N) BACE-1 protein expression CTRL = 5, MetS = 5, MetS + met = 5; APP protein expression in the hippocampus CTRL = 4, MetS = 6, MetS + met = 5 and hypothalamus CTRL = 3, MetS = 7, MetS + met = 6; gene expression in the hippocampus CTRL = 11, MetS = 12, MetS + met = 12 and hypothalamus CTRL = 11, MetS = 12, MetS + met = 9. *p ≤ 0.05, **p ≤ 0.01.

Sucrose intake or metformin treatment did not change BACE-1 expression in the hippocampus or hypothalamus (Fig. 3A–C). APP protein expression was similar in all groups (Fig. 3D), in the hippocampus and hypothalamus.

*BACE-1* gene expression was augmented in the hippocampus and hypothalamus of rats with MetS (Fig. 3E). Metformin increased *BACE-1* gene expression relative to controls in the hippocampus, while it had no effect in the hypothalamus. *APP* gene expression was increased by sucrose intake in the hippocampus, but not the hypothalamus (Fig. 3F). Metformin augmented *APP* gene expression in the hippocampus and hypothalamus.

### Effect of MetS and AMPK activation on OS markers

In order to explore the role of the MetS in generating OS we assessed some oxidative markers in the serum, hippocampus and hypothalamus. Malondialdehyde (MDA) levels were measured, by using thiobarbituric acid reactive substances (TBARS), to determine lipid peroxidation. The antioxidant capacity was evaluated by the effect of sample antioxidants on the oxidation of ABTS [2,2′-azino-bis(3-ethylbenzothiazoline-6-sulphonic acid)].

**Figure 4:**
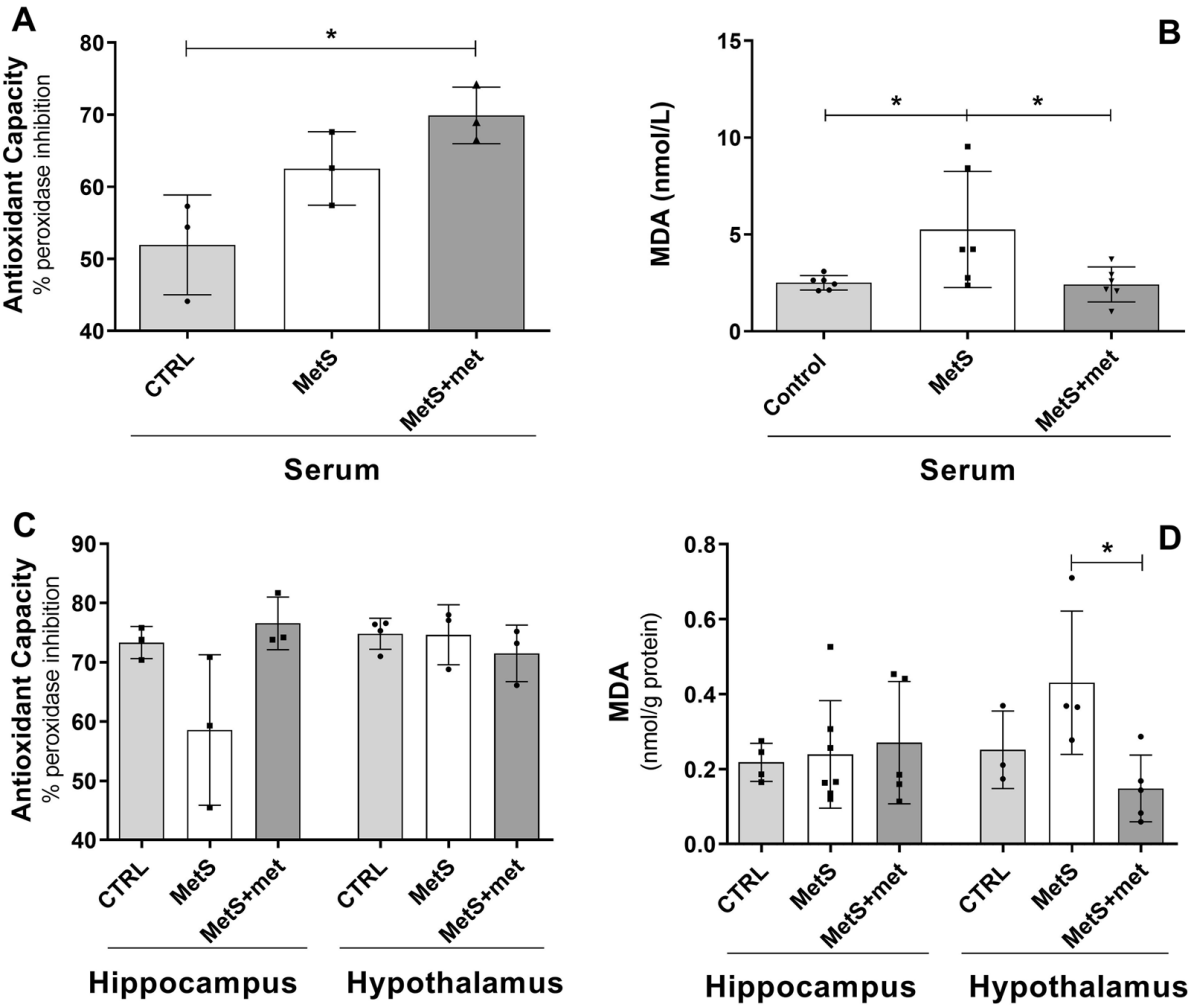
Effect of the metabolic syndrome (MetS) and metformin on oxidative stress. (**A**, **B**) Serum antioxidant capacity and lipid peroxidation. (**C**, **D**) Brain tissue (hippocampus, hypothalamus) antioxidant capacity and lipid peroxidation. The data represent the mean ± SD. Sample size (N) serum and hippocampal antioxidant capacity CTRL = 3, MetS = 3, MetS + met = 3; for serum lipid peroxidation CTRL = 6, MetS = 6, MetS + met = 6; hypothalamic antioxidant capacity CTRL = 4, MetS = 3, MetS + met = 3; hippocampal lipid peroxidation CTRL = 4, MetS = 7, MetS + met = 5; hypothalamic lipid peroxidation CTRL = 3, MetS = 4, MetS + met = 5. *p ≤ 0.05.

Serum antioxidant capacity was increased by metformin treatment compared to controls (Fig. 4A). Animals with MetS showed higher serum lipid peroxidation than controls, metformin reverted lipid peroxidation back to control levels (Fig. 4B). The antioxidant capacity was similar between the groups in the hippocampus and hypothalamus (Fig. 4C). The groups also showed similar lipid peroxidation in the hippocampus (Fig. 4D), but metformin reduced lipid peroxidation in the hypothalamus compared to animals with MetS.

### Effect of MetS and AMPK activation on the activity of antioxidant enzymes

**Figure 5:**
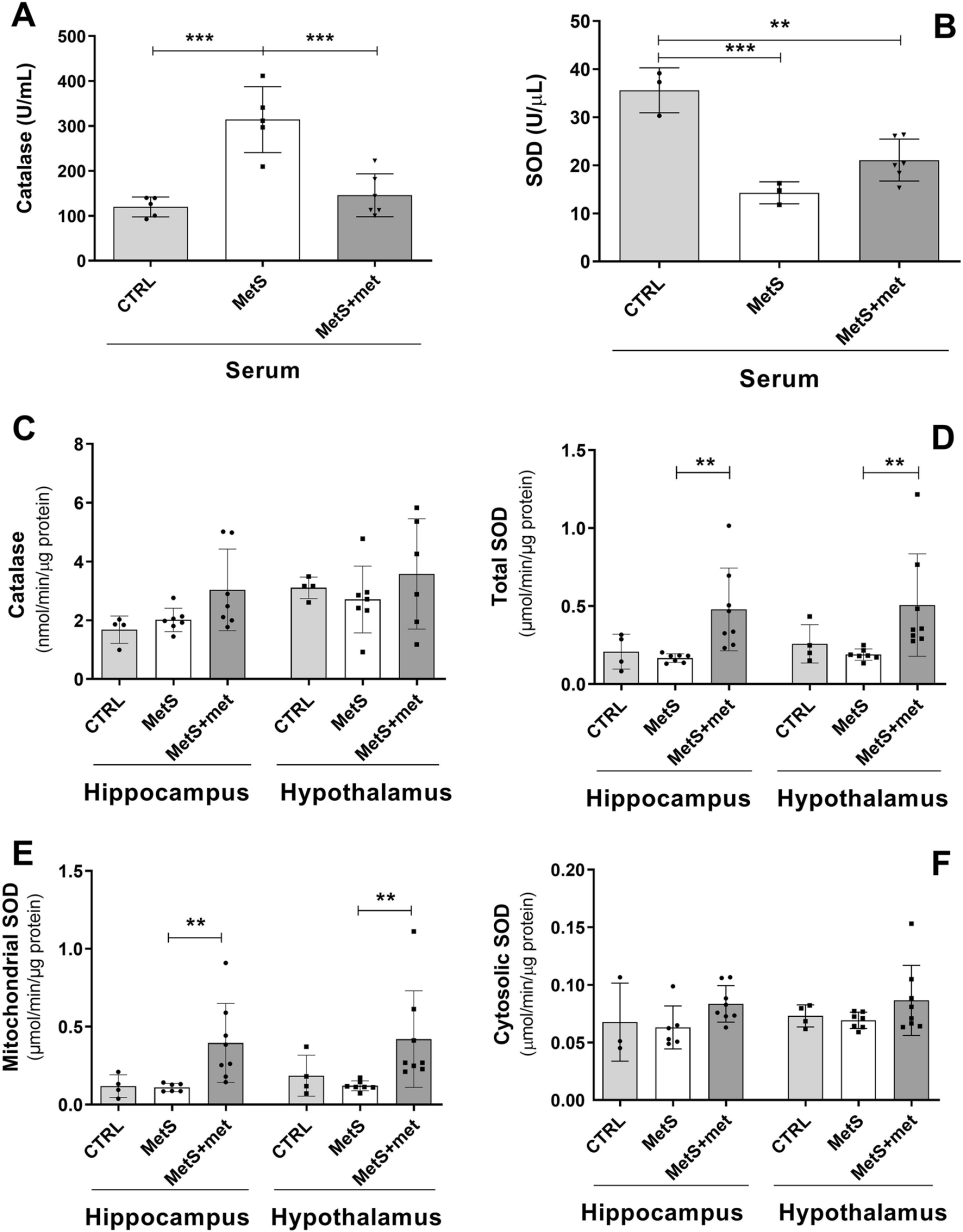
Effect of the metabolic syndrome (MetS) and metformin on antioxidant enzyme activity. (**A**, **B**) Serum catalase and superoxide dismutase (SOD) activity. (**C**) Brain catalase activity. (**D–F**) Total, mitochondrial and cytosolic SOD activity in the hippocampus and hypothalamus. The data represent the mean ± SD. Sample size (N) serum catalase CTRL = 5, MetS = 5, MetS + met = 6; serum SOD CTRL = 3, MetS = 3, MetS + met = 6; hippocampal catalase CTRL = 4, MetS = 7, MetS + met = 7; hypothalamic catalase CTRL = 4, MetS = 7, MetS + met = 6; total SOD, hypothalamic cytosolic and mitochondrial SOD CTRL = 4, MetS = 7, MetS + met = 8; hippocampal cytosolic SOD CTRL = 3, MetS = 6, MetS + met = 8; hippocampal mitochondrial SOD CTRL = 4, MetS = 6, MetS + met = 8. **p ≤ 0.01, ***p ≤ 0.001.

Serum catalase (CAT) activity was augmented by sucrose intake relative to controls and reduced back to control levels by metformin (Fig. 5A). In contrast, serum SOD activity was decreased in rats with MetS and in metformin-treated animals compared to controls (Fig. 5B). CAT activity in the hippocampus and hypothalamus was similar between the groups (Fig. 5C). Total SOD activity was increased by metformin treatment compared to rats with MetS in both brain areas (Fig. 5D). This was mainly due to mitochondrial SOD rather than cytosolic SOD, since the same differences were found for the former and no changes were observed for the latter (Fig. 5E, F).

### Effect of MetS and AMPK activation on creatine kinase

**Figure 6:**
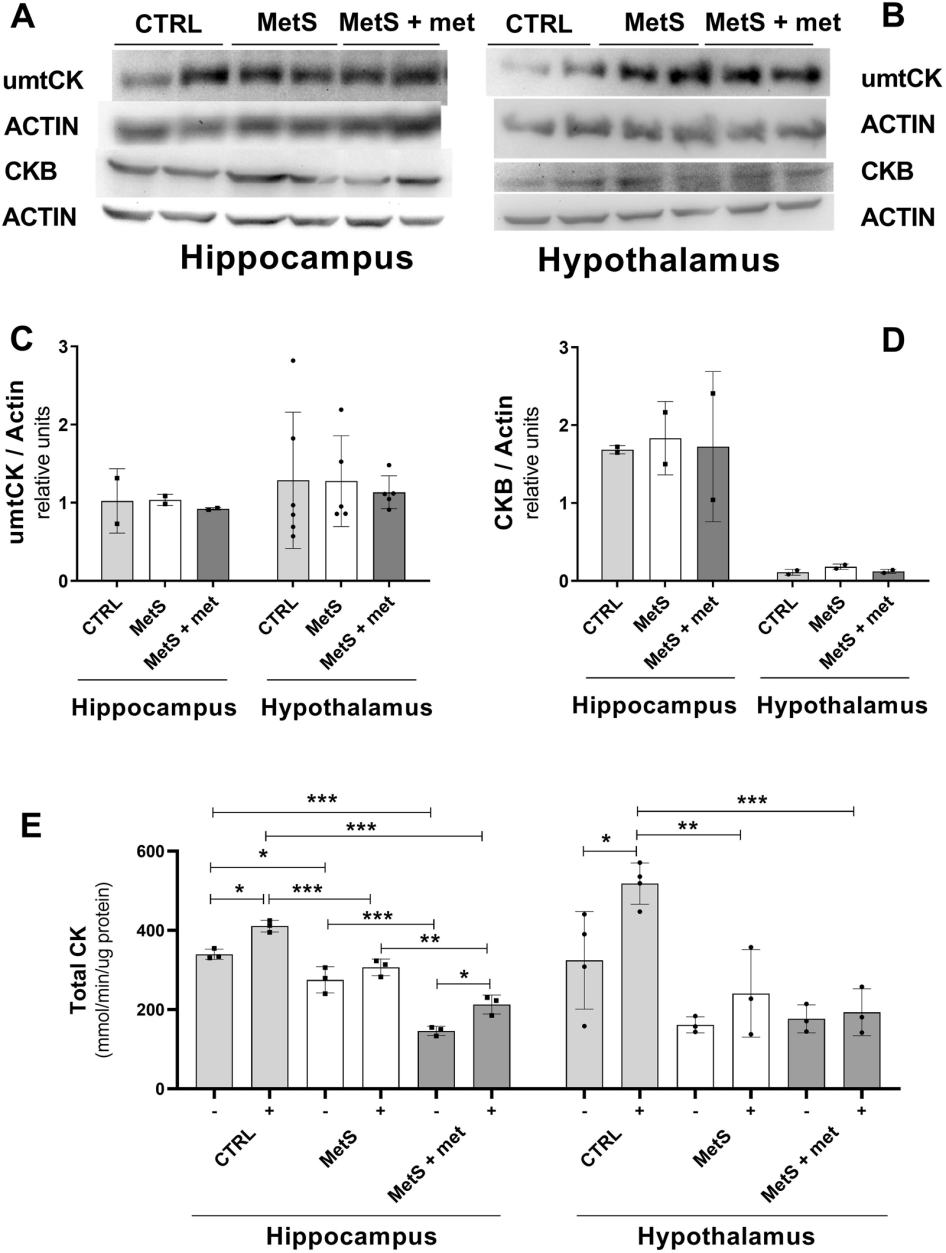
Effect of the metabolic syndrome (MetS) and metformin on ubiquitous mitochondrial (umtCK) and brain creatine kinase (CKB) expression as well as total creatine kinase (CK) activity. (**A**, **B**) Representative blots showing umtCK expression in the hippocampus and hypothalamus. (**C**, **D**) Densitometric analysis of umtCK and CKB expression in the hippocampus and hypothalamus (**E**) Total CK activity with or without dithiothreitol (DTT) in the hippocampus and hypothalamus. The data represent the mean ± SD. Sample size (N) CKB and hippocampal umtCK expression CTRL = 2, MetS = 2, MetS + met = 2; hypothalamic umtCK expression CTRL = 6, MetS = 2, MetS + met = 5; total CK activity with or without DTT in the hippocampus (all = 3) or hypothalamus (control with or without DTT = 4, all others = 3). *p ≤ 0.05, **p ≤ 0.01, ***p ≤ 0.001.

Creatine kinase (CK) is the most important element of the energy circuit that produces ATP from phosphocreatine reserves in the brain^28^ and its activity is highly sensitive to free oxygen radicals^29^. Thus, we used its enzymatic activity to evaluate oxidative damage of the energy producing machinery in the brain. We also evaluated if DTT (a reducing agent) recovered CK activity. Total CK activity was reduced in the hippocampus of rats with MetS compared to controls and it was not restored by metformin administration relative to the other groups, whether or not DTT was present in the assay mix (Fig. 6E). Animals with MetS and metformin-treated rats showed reduced total CK activity compared to controls, but only with DTT, in the hypothalamus. Additional CK activity in both brain areas of controls, but not in rats with MetS, was recovered by DTT. The data indicate that CK structure was severely damaged by OS generated in the tissues of animals with MetS, affecting its catalytic capacity rather than protein expression, since metformin did not restore it back to control values. This was verified by evaluating the expression of ubiquitous mitochondrial CK and brain CK, neither of which changed between the groups in both brain areas (Fig. 6C,D).

### Effect of MetS and AMPK activation on NFE2L2 gene expression

**Figure 7:**
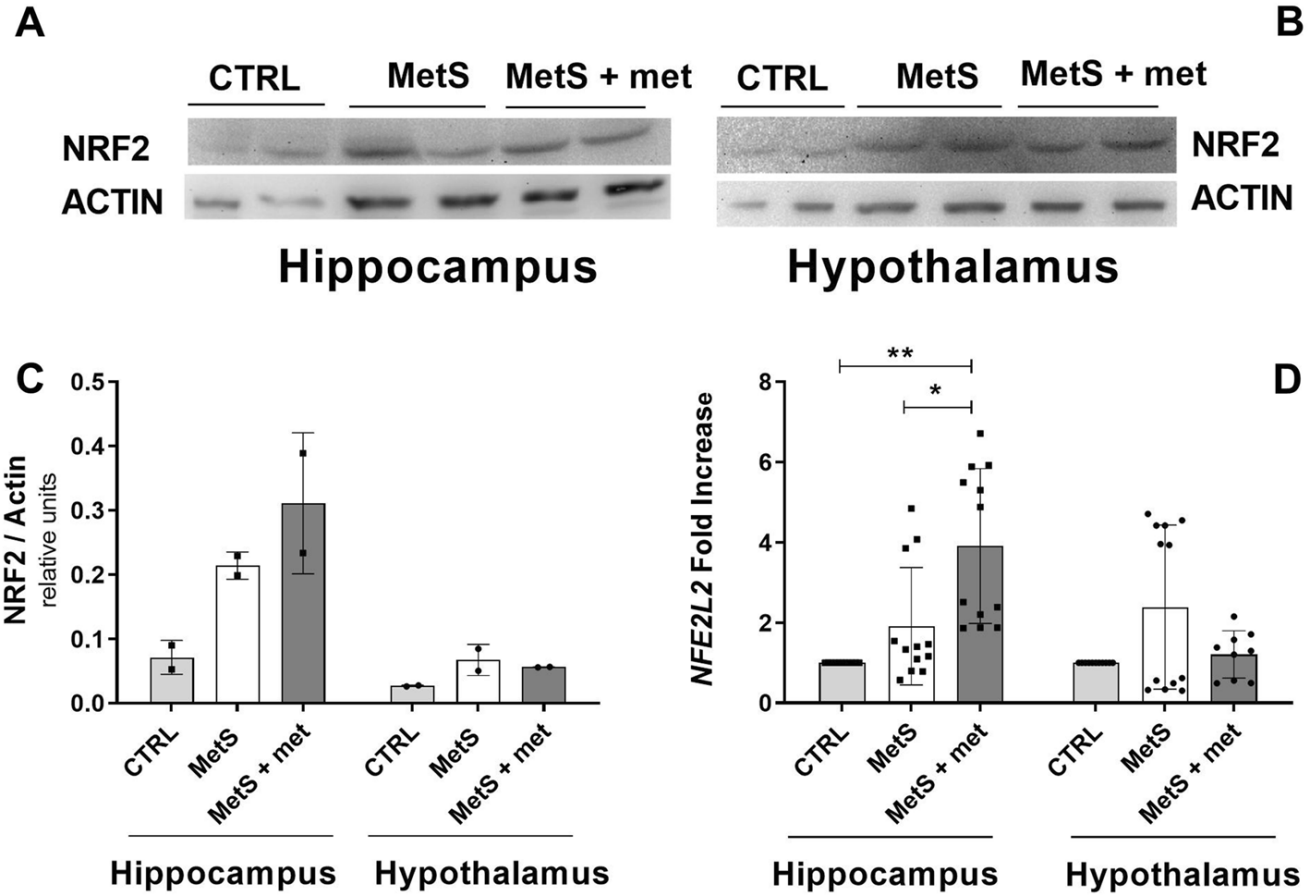
Effect of the metabolic syndrome (MetS) and metformin on NRF2 protein and *NFE2L2* gene expression in the hippocampus and hypothalamus. (**A**, **B**) Representative blots showing NRF2 protein expression in the hippocampus and hypothalamus. (**C**) Densitometric analysis of NRF2 protein expression in the hippocampus and hypothalamus. (**D**) *NFE2L2* gene expression in the hippocampus and hypothalamus. The data represent the mean ± SD. Sample size (N) for NRF1 protein expression CTRL = 2, MetS = 2, MetS + met = 2; hippocampal *NFE2L2* gene expression CTRL = 11, MetS = 12, MetS + met = 12; hypothalamic *NFE2L2* gene expression CTRL = 11, MetS = 12, MetS + met = 9. *p ≤ 0.05, **p ≤ 0.01.

Nuclear factor erythroid 2-related factor 2 or nuclear factor erythroid-derived 2-like 2 (NRF2 or NFE2L2) transcription factor is a target of activated AMPK that increases the expression of antioxidant enzymes^30^. NRF2 protein expression was similar between the groups in both brain areas (Fig. 7A,B). *NFE2L2* gene expression was increased by metformin treatment compared to both other groups in the hippocampus (Fig. 7D). *NFE2L2* gene expression was similar between the groups in the hypothalamus (Fig. 7C).

## Discussion

Several studies have related dementia and neurodegenerative processes to metabolic disorders. In this sense, MetS is a risk factor that accelerates neurodegenerative diseases such as AD^5^. Notably, the elevation of reactive oxygen species (ROS) leading to OS in MetS causes injury to neurons^31^. This work attempted to better understand the cellular mechanisms that link MetS to neurodegeneration. We focused on the role of excessive ROS and AMPK-mediated alleviation by metformin treatment.

As previously reported, 16 weeks of a high sucrose-diet altered metabolic markers in rats^26,32^, validating this diet as a model of MetS. The lack of changes in glucose, body weight or arterial pressure suggests incipient MetS development^26^. In this line, serum OS markers imply incipient MetS since the antioxidative capacity was similar to controls. MDA was a more sensitive biomarker of MetS, since it was increased in the sucrose-fed group. It has been shown that serum lipid peroxidation increases proportionally to the duration of the high sucrose-diet^25,33^. Animals subjected to a high sucrose diet have reduced antioxidant enzymes, such as CAT and superoxide^34^. The results show that this is true in serum, even when the antioxidative capacity is normal. In this sense, the model used may unravel the early mechanisms leading to severe MetS co-morbidities; such as OS-onset likely generated by a decrease in the antioxidant machinery. Moreover, the data suggest that important cellular damage occurs at this early stage of MetS development. The results also support a beneficial effect of metformin on the metabolic status of rats with MetS (insulin, glucose and HOMA), probably due to AMPK activation. In this line, dose-dependent effects of metformin have been reported^35–37^. Thus, it is likely that its effects would have been more pronounced if we had used a higher dose. We chose this dose since it was not expected to affect insulin levels^38^.

The hippocampus and hypothalamus responded differently to MetS. The results suggest insulin resistance in the hypothalamus due to the metabolic condition induced by the sucrose diet, since no changes were observed in the insulin and AMPK cascades despite higher insulin levels. It is known that chronic metformin administration increases AMPK activation^39^; indeed, this was the case in the hippocampus and hypothalamus. AMPK phosphorylation was unchanged by sucrose intake. Decreased AMPK expression might partly explain this finding.

AD neurodegeneration is closely related to cell lesions involving amyloid peptides, which come from selective APP cleavage by BACE-1. BACE-1 and APP protein expression were unchanged in MetS animals. BACE-1 and APP gene expression increased in both brain areas of MetS rats. In the hypothalamus, metformin restored APP gene expression back to control levels. Metformin treatment augmented APP and BACE-1 genes but not protein expression. The changes in BACE-1 gene expression might be explained by alterations in BACE-1 activity by modulators; such as OS, which may enhance its catalytic activity^40^. Metformin treatment increased BACE-1 gene expression in the hippocampus of MetS rats. When Aβ processing is significant, OS generated by MetS may enhance BACE-1 activity and concomitant APP cleavage to Aβ peptides, which might result in plaques^41,42^. However, Aβ 40–42 peptides were not detected likely because of their low expression in the rat brain. AMPK activation may reduce Aβ formation by affecting BACE-1 expression.

On the other hand, APP cleavage by BACE-1 into Aβ may enhance ROS production^13,14^. In this sense, sucrose intake augmented BACE-1 and APP gene expression as well as worsening OS. This suggests a contribution of APP flux through BACE-1 in generating OS during development of MetS. No doubt that BACE-1 activity plays a pivotal role in these processes and could be determined by using our model in the future.

Sucrose consumption augmented serum lipid peroxidation. Metformin treatment increased serum antioxidant capacity while restoring lipid peroxidation, by activating AMPK or inhibiting complex I of the respiratory chain. Indeed, this early MetS stage likely evoked a compensatory response in serum catalase activity. IR has been proposed to induce a low energy cellular condition, which is sensed by AMPK by increasing its phosphorylation. However, when IR persists or worsens, the compensatory response may be insufficient and other co-morbidities appear. Such results have been shown before; for example the AMPK pathway is unchanged or even blunted during late onset of T2D^43^.

The hippocampus and hypothalamus responded differently to metformin treatment, probably indicating a compensatory response to surmount the nascent insulin unresponsiveness. This was suggested before in a murine model over-expressing human APP, which develops IR and has enhanced AMPK activation together with an energy unbalance^33^. Controversially, some reports showed that high-fat diets did not induce changes in AMPK content or activation^44^. However, these diets rapidly induce hyperglucemia and accelerate the onset of other MetS traits, indicating advanced disease development. In contrast, our results show a transition phase in AMPK content, where its down-regulation is a result of the metabolic disturbances imposed by high-sucrose feeding, at least in the hippocampus.

CK activity was greatly reduced by incipient MetS in both the hippocampus and hypothalamus. Moreover, neither a reducing agent (DTT) nor metformin treatment augmented its activity in MetS animals. Thus, OS likely is present at early stages of all neurodegenerative illnesses, causing irreversible damage to CK structure and activity. This may explain why creatine supplementation lacks beneficial effects on neurodegenerative diseases^45^, does not protect neurons from Aβ-induced apoptosis and worsens cognition in a rat model of AD^46^. However, variables such as age, diet, tissue type and creatine bioavailability may also influence the negative outcomes of creatine supplementation seen so far^47–50^.

NEF2L2 may be part of the AMPK signaling pathway, mediating the ROS response^51^. Indeed, we found that AMPK activation elicited by metformin treatment causes an antioxidative response in both brain areas, reflected by augmented systemic antioxidant capacity. Moreover, SOD over-expression in both brain areas was likely due to *NFE2L2* gene activation induced by metformin treatment. Indeed, the antioxidative effects of AMPK-NFE2L2 on cognitive function likely play an importante role in the protective effects of resveratrol^52^.

The insulin/PKB axis was increased by metformin, indicating that under the remodeled redox status imposed by MetS, this signaling pathway responds in a different way from canonical models^53^. This may be explained by AMPK activation increasing antioxidative defenses, which in turn inhibit phosphatase acivity augmenting phosphorylation of PKB. On the other hand, an AMPK-independent PKB response to the incipient IR may be responsible for overactivation of the insulin cascade.

## Conclusions

Our results evidenced that at early stages in the development of MetS, metabolic disturbances in the brain affect energy pathways such as the insulin cascade. OS is present at the systemic level and in the brain; it may initially trigger an antioxidative response to reduce oxidative damage. Moreover, the altered metabolic environment favors amyloidgenesis by enhancing BACE-1 and APP gene expression, contributing to neurodegenerative processes. AMPK activation may help to restore the redox state of the brain through NEF2L2. The relevance of such activation on systemic oxidative damage and its cognitive consequences remain unknown. Further studies are needed to explore the therapeutic potential of antioxidative therapies and AMPK activation as preventive treatments during premature stages of MetS.

## Methods

The institutional committees for the use and care of animals and for ethics in research of our institute approved all procedures (protocol number INP 025/2015), which were in accordance with national (NOM-062-ZOO-1999) and international (NIH guide for the care and use of experimental animals) standards. The study was performed in compliance with the ARRIVE guidelines^54^. All reagents, unless otherwise specified, were purchased from Sigma-Aldrich Corporation.

### Animals and groups

We purchased 44 male Wistar rats (Instituto de Investigaciones Biomédicas, UNAM) weighing 250–300 g (~ 8–9 weeks old) initially. Small groups of rats (2–4) were housed in plexiglass cages (36 × 46 × 20 cm) within a barrier animal facility. The bedding material was oak sawdust, it was changed 3 times per week. Controlled conditions of temperature (24 ± 2 °C), humidity (40–60%) and lighting (12:12 cycle, lights on at 6 AM) were used throughout. Any animal showing signs of pain, discomfort or disease (other than MetS proper) were to be excluded, but none met the previously established criteria.

The sample size was based on previous publications^26^. No randomization was done because the rats are highly homogenous. 28 animals received 30% sucrose in their drinking water during 16 weeks to induce MetS, the other 16 rats (controls) drank normal water ad libitum. The drinking water was changed 3 times per week. All animals were fed normal rat chow (5008, LabDiet) and weighed weekly. At the end of week 16, serum triglyceride levels were measured after a 6-h fast. As expected, control rats showed < 200 mg/dL and MetS animals had > 200 mg/dL serum triglyceride levels (inclusion criteria for the groups). Control rats were euthanized, MetS animals were subdivided and maintained for another 6 weeks: 16 rats only received 30% sucrose, 12 received 30% sucrose and metformin (100 mg/kg/day, p. o.). Their serum triglyceride levels were measured again after this period. Blinding of participants was not possible during treatment, since the rats/groups were clearly distinguishable and was not practical afterwards to minimize human error. Although other potential confounders (such as treatment/ measurement order, animal/cage location) were not controlled, none could significantly alter the results obtained. What follows are the outcome measures that were assessed.

### Tissue processing

The number of animals used for each experiment is indicated in the figure legends. Blood was obtained and centrifuged at 3000 rpm for 15 min at 4 °C to obtain the serum, which was stored at − 70 °C. The rats were sacrificed with an over-dose (200 mg/kg) of sodium pentobarbital (Pisabental, Pisa). The hippocampus and hypothalamus were rapidly dissected, separated and cut into two portions. One portion was used for RNA isolation and PCR, while the other was used for protein expression and enzymatic activity. For the latter, the tissue was homogenized at 4 °C with a lysis buffer containing (mM): 50 HEPES, 1 EGTA, 50 KCl, 50 NaF, 5 NaPPi, 0.2 PMSF, 1 NaVO_4_ and 0.1% Triton X-100 plus protease inhibitors (pH 7.4). After 30 min of incubation at 4 °C, the homogenate was centrifuged at 12,000×*g* for 30 min at 4 °C and the supernatant was recovered. Samples were aliquoted and stored at − 70 °C for further use. Additionally, the retroperitoneal fat pads were obtained and weighed as a measure of abdominal fat.

### Blood and plasma measurements

Blood triglyceride levels were measured after a 6-h fast with a digital monitor system (Accutrend, Roche). Similarly, blood glucose levels were measured after a 6-h fast with a digital monitor system (OptiumXceed, Abbott Laboratories). Serum insulin was determined by enzyme linked immunosorbent assay (Alpco Diagnostics). The HOMA index was calculated from glucose and insulin measurements, using the standard equation ^55^.

### Western blotting

The hippocampus and hypothalamus were obtained and homogenized at 4 °C, with the same lysis buffer mentioned above plus DTT (1 mM). Protein concentration was determined by the Lowry method^56^. Total protein (50 μg) was separated in 10% acrylamide gels using SDS-PAGE, transferred to PVDF membranes and probed with: phospho-AMPKα (Thr172), AMPKα, phospho-PKB (Ser473) (2535, 2532, 9018, Cell Signaling Inc.), PKB/AKT (610860, BD Transduction Laboratories), APP, BACE-1 (ab126873, ab2077, Abcam), umtCK (sc-15166, Santa Cruz Biotechnology Inc.) and NRF2 (ADI-KAP-TF125-D, Enzo). Membranes were revealed using chemiluminescence assays and the amount of total protein was analyzed using Quantity-One software (Bio-Rad Laboratories Inc.), images were cropped to improve the clarity of the bands, full-length blots are presented in the Supplementary Figures. Actin was detected with an antibody (A2103, SIGMA) and used as a loading control.

### Lipid peroxidation

MDA was quantified by derivatization with TBARS as previously published^25^. Brain tissues (0.4 mg) or plasma (200 μL) were treated with 4% butylhydroxytoluene (MP Biomedicals) to prevent further oxidation. Phosphate buffer (pH 7.4) was added and the samples were incubated for 30 min at 37 °C under constant agitation. TBARS (0.8%) and 20% acetic acid (pH 3.5) were added and the mixture was incubated for 1 h at 100 °C. Placing the mixture in ice stopped the reaction; afterwards 2% KCl and 5 mL *n*-butanol were added to extract lipids. The organic phase was obtained and fluorescence was determined at 515/553 nm excitation/emission (Modulus Microplate, Promega Corporation), using tetraethoxypropane as a calibrating standard.

### Total antioxidant capacity

Brain tissues (150 µg) or serum (50 µL) were added to a mixture (1 mL) of 20 µM ABTS, 25 IU peroxidase and 200 µM H_2_O_2_ diluted in phosphate buffer (pH 7). Oxidized ABTS was monitored by measuring absorbance at 414 nm with a UV–VIS spectrophotometer (CARY 100, Agilent Technologies).

### Antioxidant enzyme activities

Catalase and superoxide dismutase (SOD) activities were determined by fluorometric and spectrophotometric enzymatic methods using commercial kits (ADI-907-027 and ADI900-157, Enzo Life Sciences, Inc). The hippocampus and hypothalamus were obtained and homogenized at 4 °C with the same lysis buffer mentioned above (without DTT). All other procedures were done according to the manufacturer´s instructions. Mitochondrial SOD was identified by adding 1 mM sodium azide to the reaction mixture.

### CK activity

A system of enzymatic-coupled reactions was used: 0.15 M sodium phosphocreatine plus 2 mM ADP were used to form ATP. 20 mM glucose and 3 µg hexokinase were added to produce glucose-6-phosphate. 15 mU glucose-6-phosphate dehydrogenase plus 2 mM NADP were used to form NADPH, which was monitored by measuring the absorbance at 340 nm (CARY 100, Agilent Technologies). Additionally, 1 mM dithiothreitol (DTT) was added to evaluate if CK activity was recovered with this reducing agent.

### RNA isolation, reverse transcription and real time PCR

Total RNA was isolated from the hypothalamus or hippocampus with RNeasy mini kits (Qiagen). cDNA was obtained following the SuperScript III kit protocol (Invitrogen, Thermo Fisher). Real time PCR was carried out using an ABI 7500 real-time PCR system (Applied Biosystems) with the following TaqMan probes: *BACE-1* (Rn00569988), *APP* (Rn00570673) and *NFE2L2* (Rn00582415_m1).

### Statistical analysis

Each animal was considered an experimental unit, since a rather small number of them were housed together (i.e. they all had equal access to food and water). If the data followed a normal distribution and showed equal variance (determined with Shapiro–Wilk and Levene tests, respectively), one-way analyses of variance (ANOVA) followed by Tukey tests were used. If the data failed either test, it was examined using Kruskal–Wallis one-way ANOVA on ranks followed by Dunn´s tests (since sample sizes were rarely the same). All statistical tests were done using SigmaPlot for Windows (Version 12.2.0.45). A p ≤ 0.05 was considered significant. Data in tables and figures is presented as the mean ± standard deviation.

## Supplementary Information

Supplementary Information.
