# Supplementary material for: Increased oxidative stress contributes to enhance brain amyloidogenesis and blunts energy metabolism in sucrose-fed rat: effect of AMPK activation
Source: Sci Rep. 2021 Oct 1;11:19547. doi: 10.1038/s41598-021-98983-w (PMC8486781; doi:10.1038/s41598-021-98983-w)

Fig. 2A Hippocampus

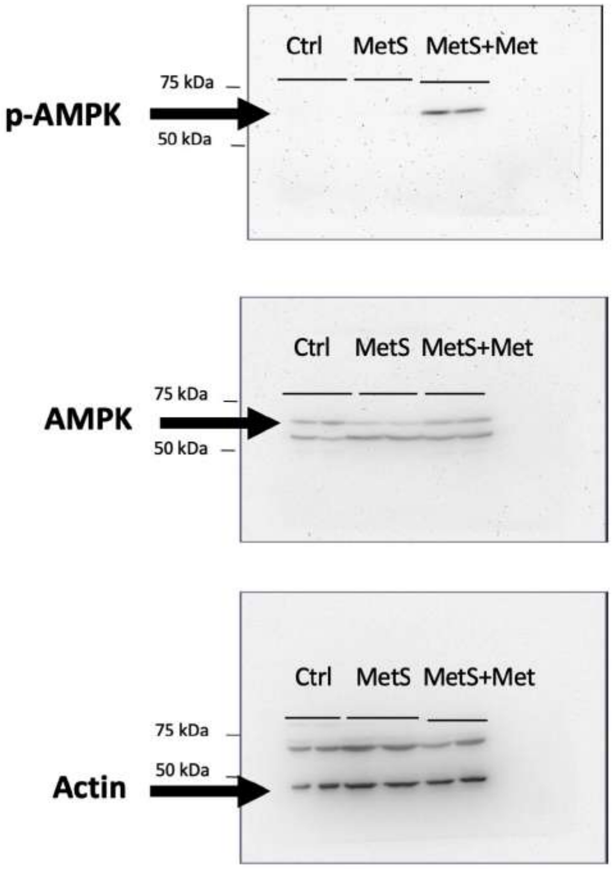

Fig. 2B Hypothalamus

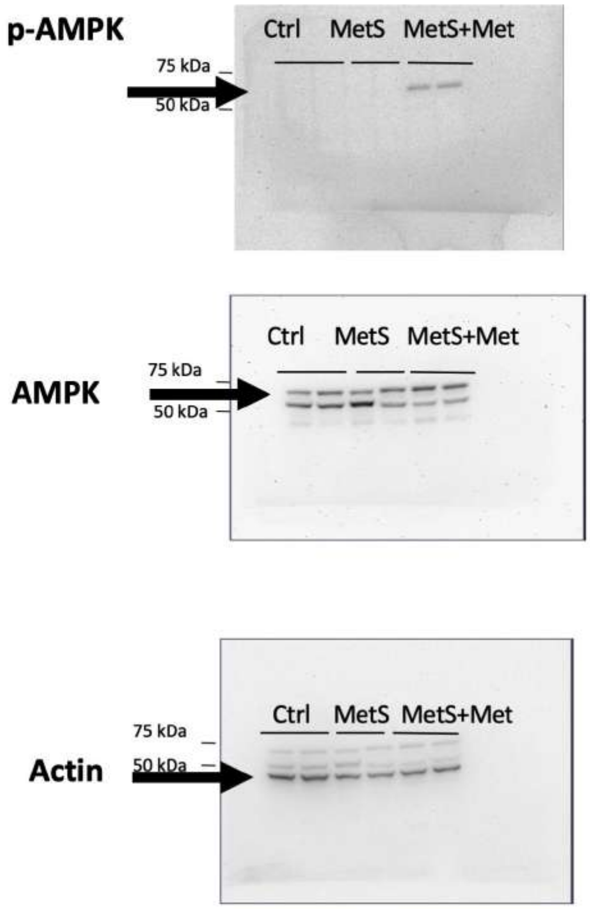

Fig. 3A Hippocampus

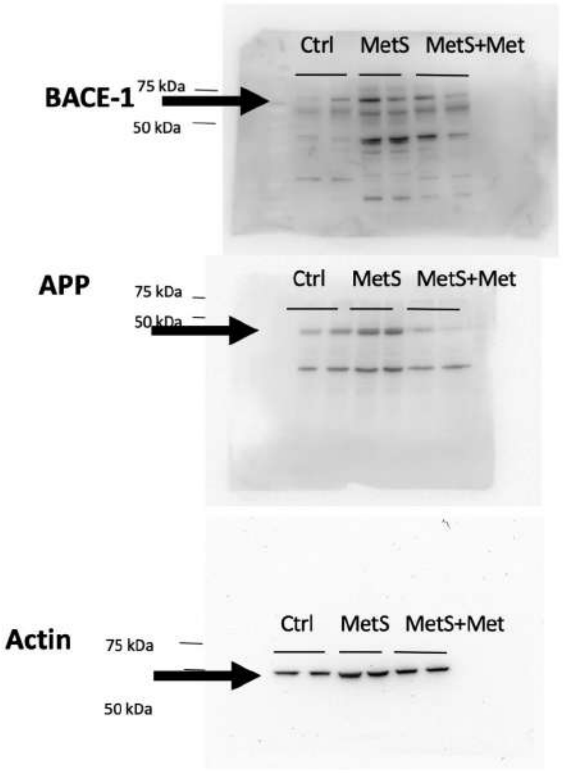

Fig. 3B Hypothalamus

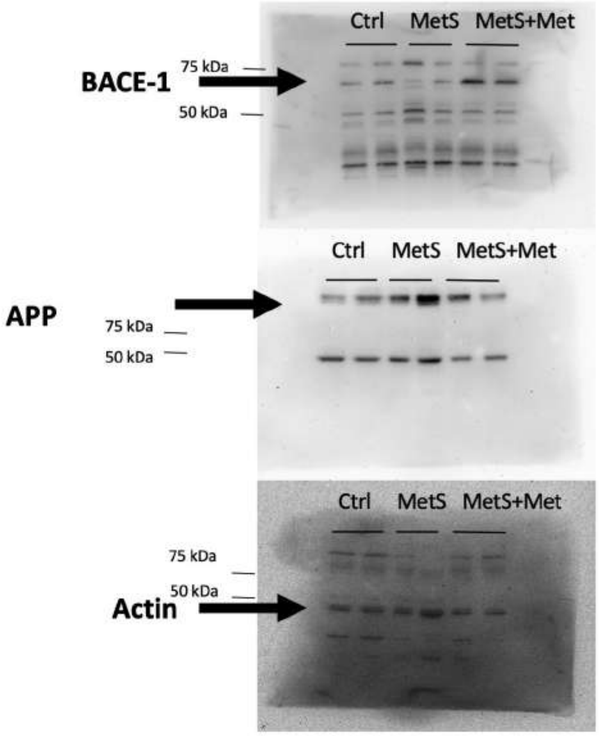

Fig. 6A Hippocampus

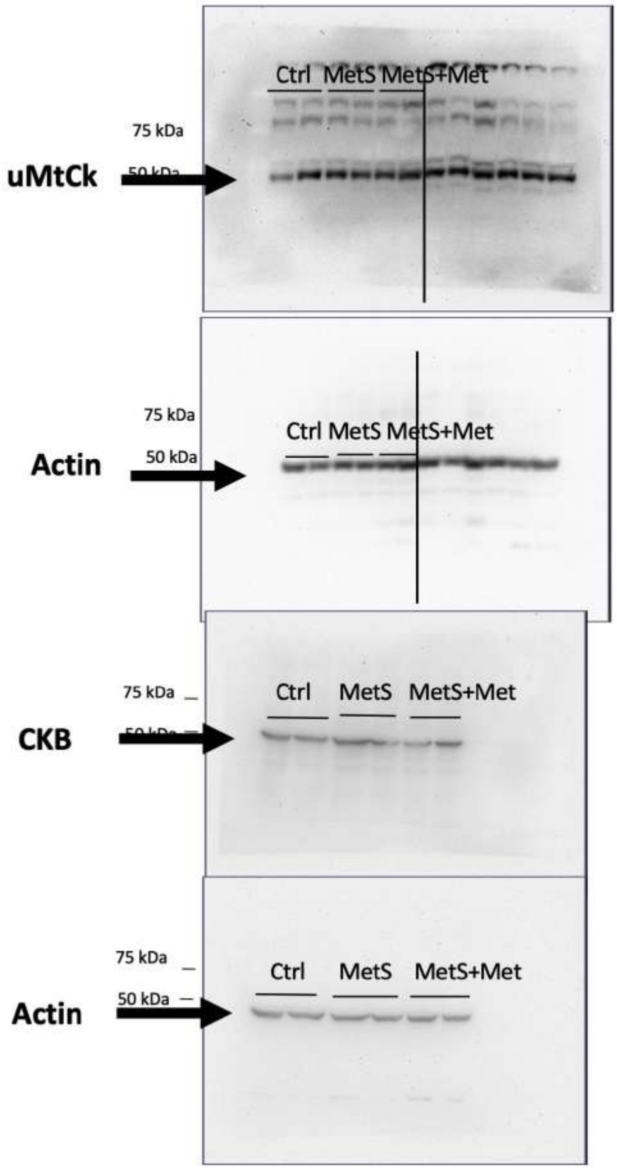

Fig. 6B Hypothalamus

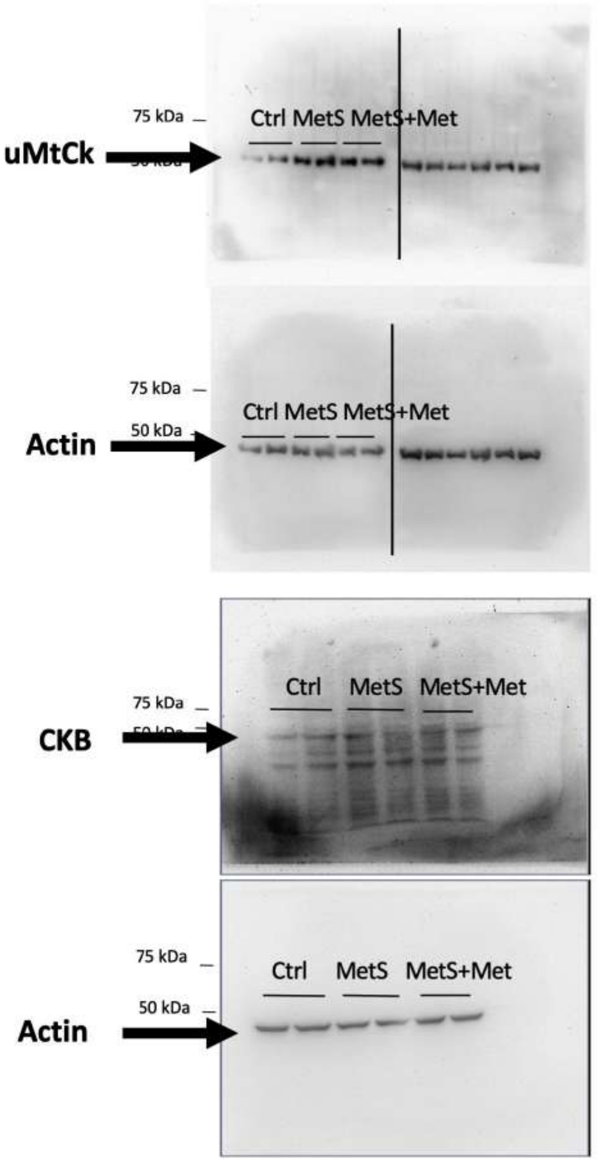

Fig. 7A Hippocampus

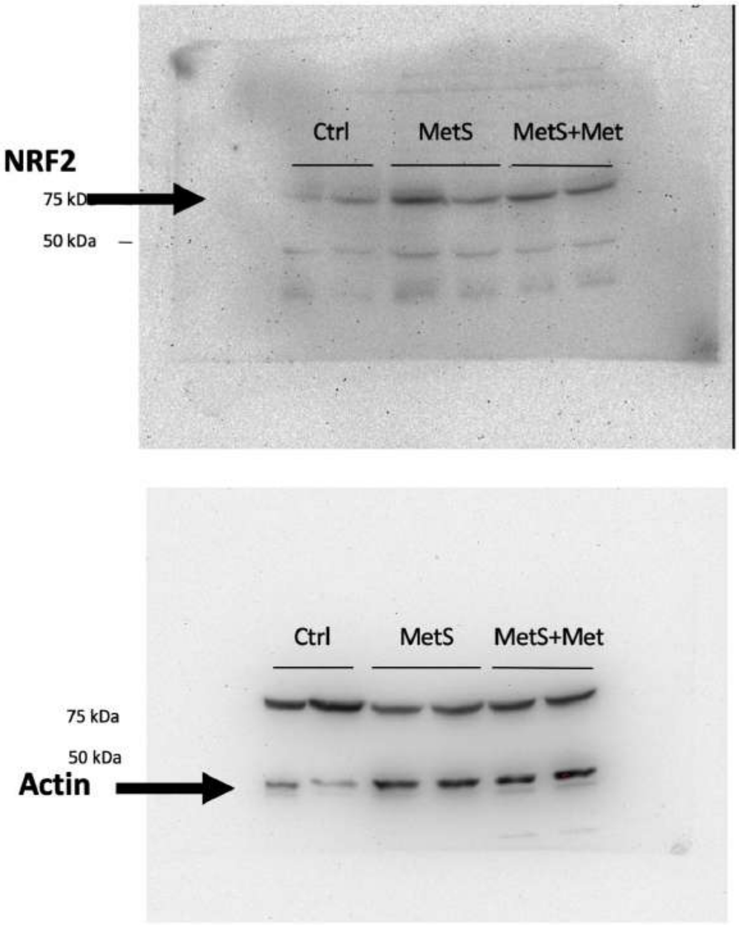

Fig. 7B Hypothalamus

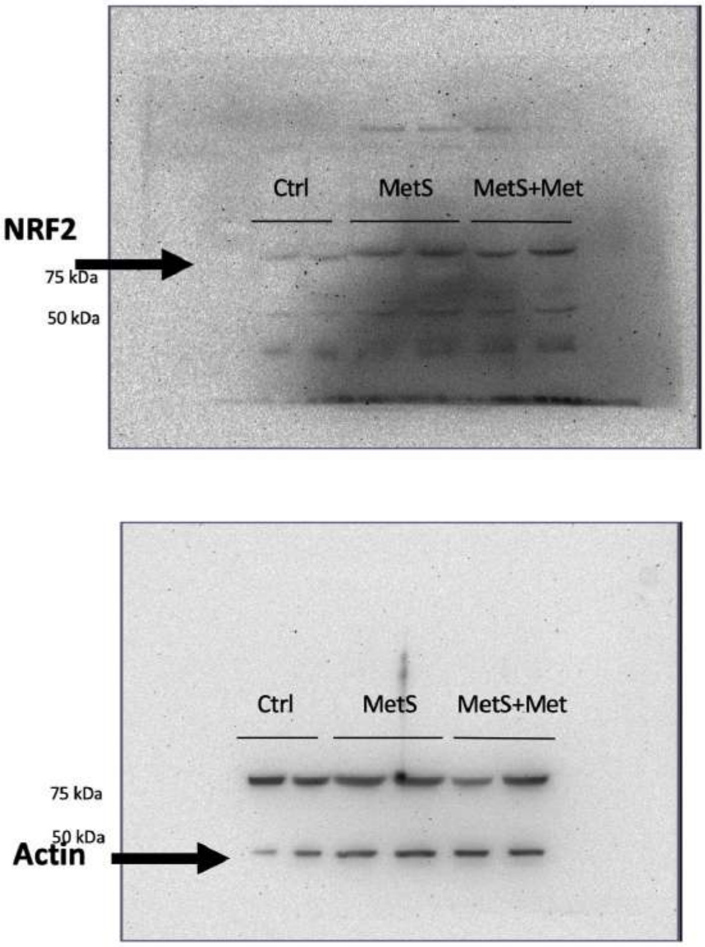

Figure 6A. Actin corresponding to CKB

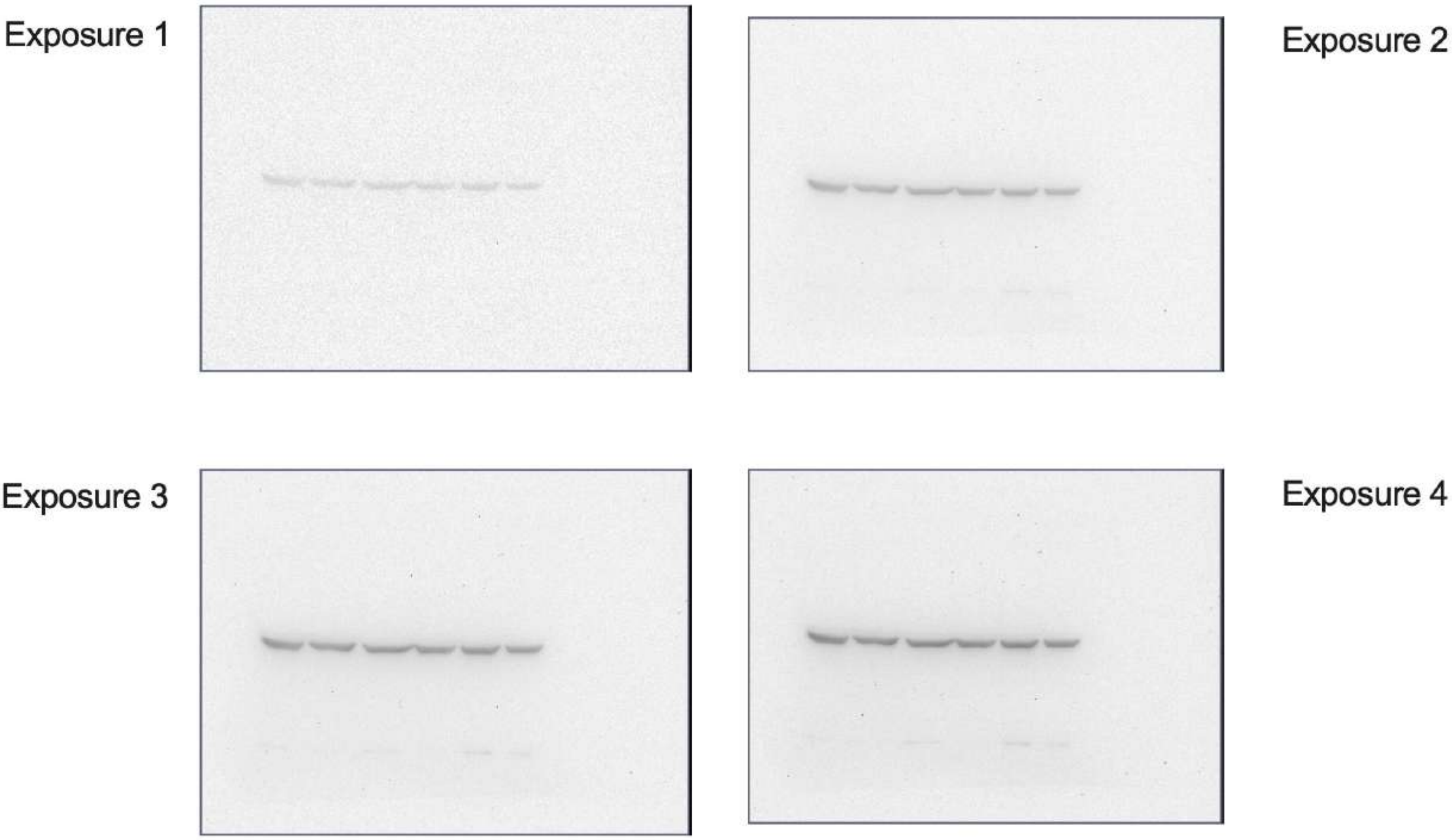

Fig. 2E Hippocampus

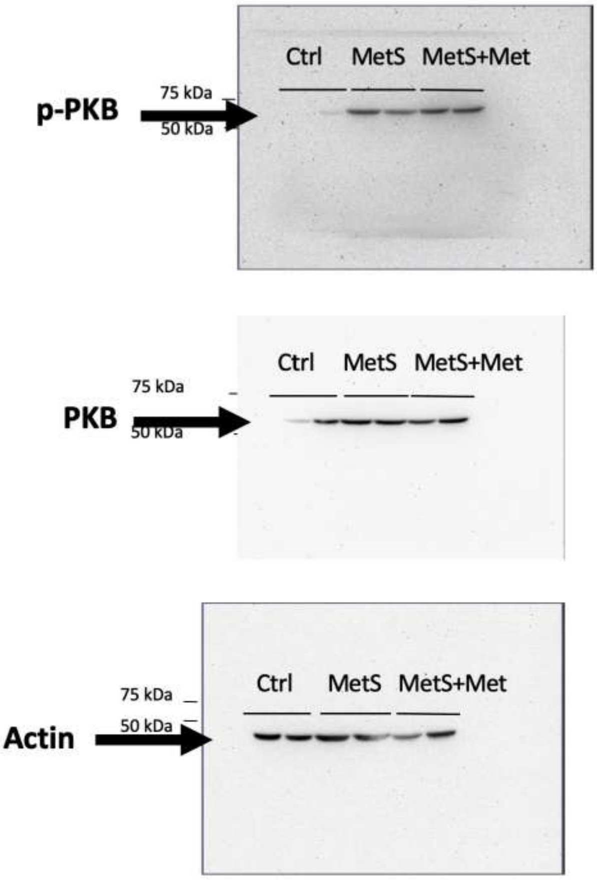

Fig. 2F Hypothalamus

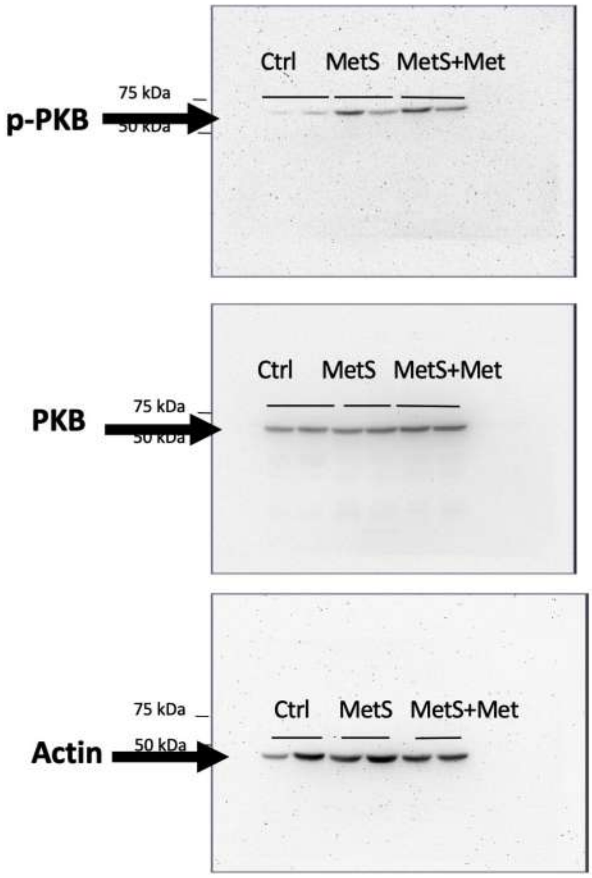

Supplement: Supplementary file 1 — Supplementary Information. [file 41598_2021_98983_MOESM1_ESM.pdf]
